# N-Octanoyl-Dopamine inhibits cytokine production in activated T-cells and diminishes MHC-class-II expression as well as adhesion molecules in IFNγ-stimulated endothelial cells

**DOI:** 10.1038/s41598-019-55983-1

**Published:** 2019-12-18

**Authors:** Björn B. Hofmann, Nicolas Krapp, Yingchun Li, Carolina De La Torre, Marloes Sol, Jana D. Braun, Matthias Kolibabka, Prama Pallavi, Bernhard K. Krämer, Benito A. Yard, Anna-Isabelle Kälsch

**Affiliations:** 1 Department of Nephrology, Endocrinology and Rheumatology, Fifth Department of Medicine, Medical Faculty Mannheim, University of Heidelberg, Mannheim, Germany; 2 Center of Medical Research, Medical Faculty Mannheim, University of Heidelberg, Mannheim, Germany; 3 Department of Medical Biology and Pathology, University Medical Center Groningen, Groningen, Netherlands

**Keywords:** Kidney, Kidney diseases

## Abstract

IFNγ enhances allograft immunogenicity and facilitates T-cell mediated rejection. This may cause interstitial fibrosis and tubular atrophy (IFTA), contributing to chronic allograft loss. We assessed if inhibition of T-cell activation by N-octanoyl dopamine (NOD) impairs adherence of activated T-cells to endothelial cells and the ability of activated T-cells to produce IFNγ. We also assessed if NOD affects IFNγ mediated gene expression in endothelial cells. The presence of NOD during T-cell activation significantly blunted their adhesion to unstimulated and cytokine stimulated HUVEC. Supernatants of these T-cells displayed significantly lower concentrations of TNFα and IFNγ and were less capable to facilitate T-cell adhesion. In the presence of NOD VLA-4 (CD49d/CD29) and LFA-1 (CD11a/CD18) expression on T-cells was reduced. NOD treatment of IFNγ stimulated HUVEC reduced the expression of MHC class II transactivator (CIITA), of MHC class II and its associated invariant chain CD74. Since IFTA is associated with T-cell mediated rejection and IFNγ to a large extent regulates immunogenicity of allografts, our current data suggest a potential clinical use of NOD in the treatment of transplant recipients. Further *in vivo* studies are warranted to confirm these *in vitro* findings and to assess the benefit of NOD on IFTA in clinically relevant models.

## Introduction

Improvements in donor management^1,2^, the continuous progress in immunosuppressive and supportive therapy^3^ as well as refinements in organ preservation^4,5^ have significantly contributed to better transplantation outcomes in the last decades. Notwithstanding the foregoing, chronic loss of organ allografts remains a major problem in contemporary transplantation medicine. Many factors can attribute to this organ loss such as T-cell and antibody mediated rejection, recurrence of the primary disease or drug toxicity^6–8^. In 2005 the term chronic allograft nephropathy has been abandoned and was redefined more histologically as interstitial fibrosis and tubular atrophy (IFTA) of unknown etiology^9^. Whilst the presence of *de novo* donor specific antibodies is an independent factor for chronic glomerulopathy, IFTA correlates much more with early T-cell mediated rejection^10^. In renal biopsies, IFTA may present with or without accompanying inflammation, i.e. i-IFTA^11^. I-IFTA is predicting disease progression, reflects active injury and typically precedes T-cell mediated rejection^12–14^. Gene expression profiling studies of renal transplant biopsies have also disclosed the association of IFTA and pathways related to T-cell activation^15^. Prevention of T-cell activation therefore is a logical rationale for preventing IFTA. Paradoxically enough, the most potent immuno-suppressive drugs that prevent T-cell activation, i.e. calcineurin inhibitors (CNI), are also a risk factor for chronic transplant loss because of their nephrotoxicity^16^.

Anti-donor immune responses are initiated in the recipient’s secondary lymphoid organs through T-cells recognition of either intact or processed donor major histocompatibility complex (MHC) molecules^17,18^. Amongst the factors that influence allograft immunogenicity, interferon gamma (IFNγ) plays a prominent role. It up-regulates the expression of MHC class I on hematopoetic and non hematopoetic cells and influences antigen presentation dendritic cells by formation of the so-called immune-proteasome (IP)^19,20^. Along with the upregulation of MHC class I also the expression of MHC class II on a large variety of non-hematopoietic cells is increased via the *de novo* expression of a specific transcription factor, i.e. MHC class II transactivator (CIITA). Immunogenicity and tissue inflammation are further facilitated by the upregulation of adhesion molecules on endothelial cells, e.g. intercellular adhesion molecule-1 (ICAM-1), vascular cell adhesion molecule -1 (VCAM-1), and production of endothelial cell derived chemokines.

We have previously reported that N-octanoyl dopamine (NOD) inhibits TNFα mediated gene expression in endothelial cells through the inhibition of a subset of nuclear factor kappa B (NFκB)-regulated genes^21^. NOD also transiently inhibits T-cell proliferation, albeit that early T-cell receptor signalling events and early intracellular cytokine concentrations were not affected by NOD^22^. In keeping with the pivotal role of IFNγ in regulating graft immunogenicity, the present study was conducted to assess if addition of NOD during T-cell activation impairs T-cell adhesion to unstimulated and IFNγ - or TNFα stimulated endothelial cells. Secondly, we sought to address if supernatants of such activated T-cells are able to induce adhesion molecules on endothelial cells and if this is paralleled by the presence of IFNγ and TNFα in the supernatants. In addition, we assessed the influence of NOD on the major ligands for adhesion molecules on T-cells, i.e. lymphocyte function associated antigen-1 (LFA-1) and very late antigen -4 (VLA4). Finally, we addressed to what extent NOD influences IFNγ mediated gene expression in endothelial cells.

## Results

### NOD impairs adhesion of activated T cells to cytokine stimulated endothelial cells

**Figure 1:**
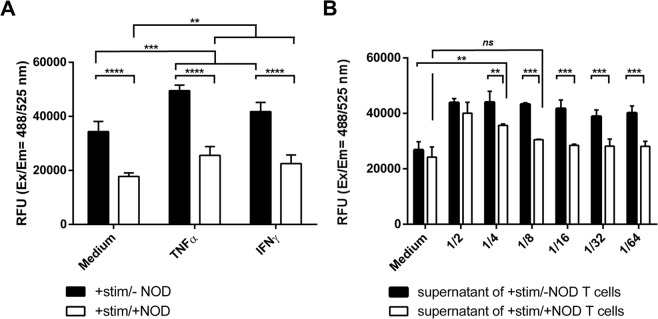
NOD impairs T-cell adhesion to endothelial cells. (**A**) T-cells were activated for 3 days by anti-CD3/anti-CD28 in the absence (filled bars) or presence (open bars) of 100 µM of NOD. Hereafter the T-cells were labelled with CytoTell green and added to unstimulated (medium) or IFNγ (50 ng/ml) or TNFα (10 ng/ml) stimulated HUVEC monolayers as described in the materials and methods. (***p ≤ 0.01; medium vs. IFNγ and TNFα, ****p ≤ 0.0001; T-cell stimulation in the absence of NOD (+stim/−NOD) vs. T-cell stimulation in the presence of NOD (+stim/+NOD)). (**B**) T-cells were activated for 3 days by anti-CD3/anti-CD28 in the absence (filled bars) or presence (open bars) of 100 µM of NOD. The supernatants were collected and added for 24 hrs in serial dilutions to endothelial cell monolayers. Hereafter the supernatants were replaced by normal medium and adhesion of activated T-cells was assessed as described above. Note that at high dilutions (1/8 and above), supernatants from T-cells that were stimulated in the presence of NOD did not significantly differ from the medium control. (**p ≤ 0.01/***p ≤ 0.001; supernatants of stimulated T-cells without NOD (+stim/−NOD) vs. supernatants of stimulated T-cells with NOD (+stim/+NOD), *ns*; not significant/**p ≤ 0.01; medium with NOD (+stim/+NOD) vs. supernatant of stimulated T-cells with NOD (+stim/+NOD)). For (**A,B**) all conditions were tested in triplicates. The results of at least 3 independent experiments are expressed as mean fluorescence + SD. Data were analysed using two-way ANOVA followed by Sidak’s multiple comparison test. A *p*-value < 0.05 was considered to be significant.

Both TNFα and IFNγ increase the expression of adhesion molecules on endothelial cells and consequently are expected to increase cell adhesion of activated T-cells to such treated monolayers of endothelial cells. As depicted in Fig. 1, anti-CD3/anti-CD28 activated T-cells indeed adhered significantly better to stimulated -, as compared to unstimulated endothelial cells. When T-cells were activated in the presence of 100 µM of NOD, T-cell adhesion was strongly diminished irrespective of treatment of the endothelial cells (Medium mean RFU ± SD: 34,387 ± 3,509 vs. 17,766 ± 1,236; *p* < 0.0001; TNFα mean RFU ± SD: 49,542 ± 1,916 vs. 25,556 ± 3,074; *p* < 0.0001; IFNγ mean RFU ± SD: 41,785 ± 3,113 vs. 22,506 ± 2,935; *p* < 0.0001; −NOD vs. +NOD). For NOD treated T-cells, adhesion to TNFα or IFNγ treated endothelial cell monolayers was slightly higher as compared to unstimulated monolayers (mean RFU ± SD: 17,766 ± 1,236 vs. 25,556 ± 3,074 vs. 22,506 ± 2,935, *p* < 0.01) (Fig. 1A).

We next assessed the propensity of supernatants obtained from anti-CD3/anti-CD28 activated T-cells to facilitate T-cell adhesion to monolayers of endothelial cells. To this end, cultured endothelial cells were stimulated overnight with serial dilution of anti-CD3/anti-CD28 activated T-cell supernatants or supernatants of T-cells activated in presence of 100 µM of NOD. While no differences in T-cell adhesion were observed between both types of supernatants in a 1 to 2 dilution, T-cell adhesion decreased to the level of the control culture medium when higher dilutions of supernatants from T-cells that were activated in the presence of NOD were used (Dilution 1/64 mean RFU ± SD: 40,180 ± 2,031 vs. 28,149 ± 1,462 vs. 26,812 ± 3,009; −NOD vs. +NOD vs. medium control *p* < 0.01) (Fig. 1B).

### VLA-4 (CD49d/CD29) and LFA-1 (CD11a/CD18) expression on T-cells are decreased by NOD

**Figure 2:**
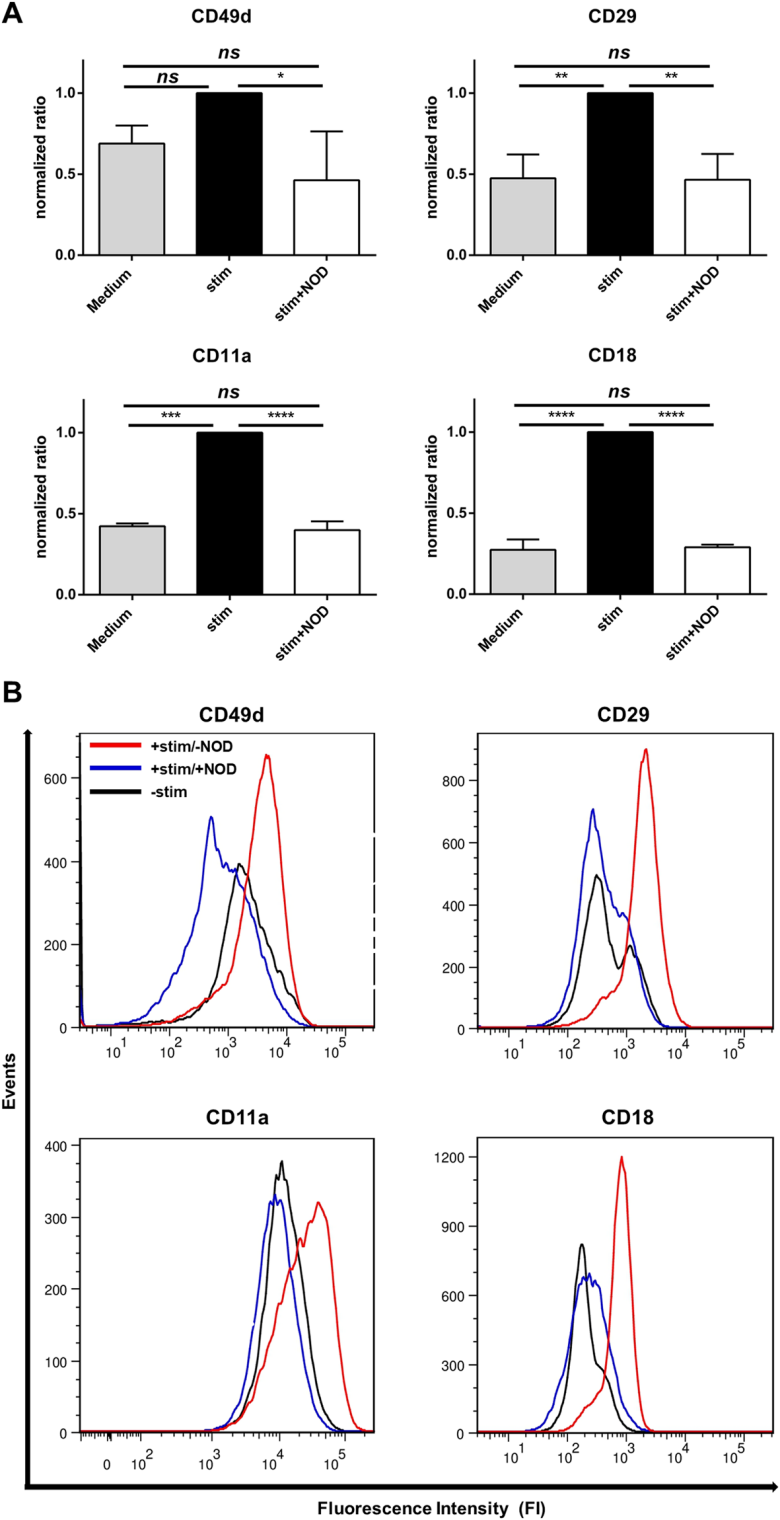
NOD inhibits VLA-4 and LFA-1 expression on activated T-cells. (**A**) The expression of VLA-4 (CD49d, CD29 upper panels) and LFA-1(CD11a, CD18 lower panels) were assessed by FACS analysis on anti-CD3/anti-CD28 activated T-cells. A total of 3 independent experiments using 3 different T-cell donors were performed. Since each donor differed in the extent by which the antigens were up-regulated following T-cell activation, the mean fluorescence intensity (MFI) was normalized for stimulation. The results are expressed as mean normalized ratio + SD, i.e. (MFI medium/MFI stimulated) or (MFI stimulated +NOD/MFI stimulated) with (MFI stimulated/MFI stimulated) as 1. (*p < 0.05; **p < 0.01; ***p < 0.001; ****p < 0.0001; ns: not significant). (**B**) The results of a representative experiment are shown as histograms. Blue histogram: T-cell activation in the presence of 100 µM of NOD. Red histogram: T-cell activation in the absence of NOD. Black histogram: VLA-4 and LFA-1 expression on unstimulated T-cells.

In keeping with the observation that T-cell adhesion to endothelial cells is diminished when T-cells are activated in the presence of NOD, we addressed if this was reflected by a reduced expression of T-cell ligands for adhesion molecules. Because the expression of ICAM-1 and VCAM-1 on endothelial cells is of pivotal importance for leucocyte extravasation, we assessed in particular if NOD influences the expression of the known ligands for VCAM-1 and ICAM-1 on T-cells, i.e. VLA-4 and LFA-1 respectively. Upon stimulation with anti-CD3/anti-CD28 both the α- (CD49d) and ß-chain (CD29) of VLA-4 were increased (CD49d MFI: 1,663 vs. 3,928; CD29 MFI: 385 vs. 1,921; unstimulated vs. anti-CD3/anti-CD28) (Fig. 2B, upper panels). Similar findings were observed for the α- and ß-chain of LFA-1, CD11a and CD18 respectively (CD11a MFI: 10,786 vs. 24,086; CD18 MFI: 119 vs. 712; unstimulated vs. anti-CD3/anti-CD28) (Fig. 2B, lower panels). Addition of 100 µM of NOD during T-cell activation completely abolished this up-regulation and decreased the expression of CD49d even below that of unstimulated T-cells (CD49d MFI: 3,928 vs. 576; CD29 MFI: 1,921 vs. 339; CD11a MFI: 24,086 vs. 8,226; CD18 MFI: 712 vs. 158; anti-CD3/anti-CD28/−NOD vs. anti-CD3/anti-CD28/+NOD) (Fig. 2B).

### Induction of endothelial VCAM-1 and ICAM-1 expression by supernatants of activated T-cells

**Figure 3:**
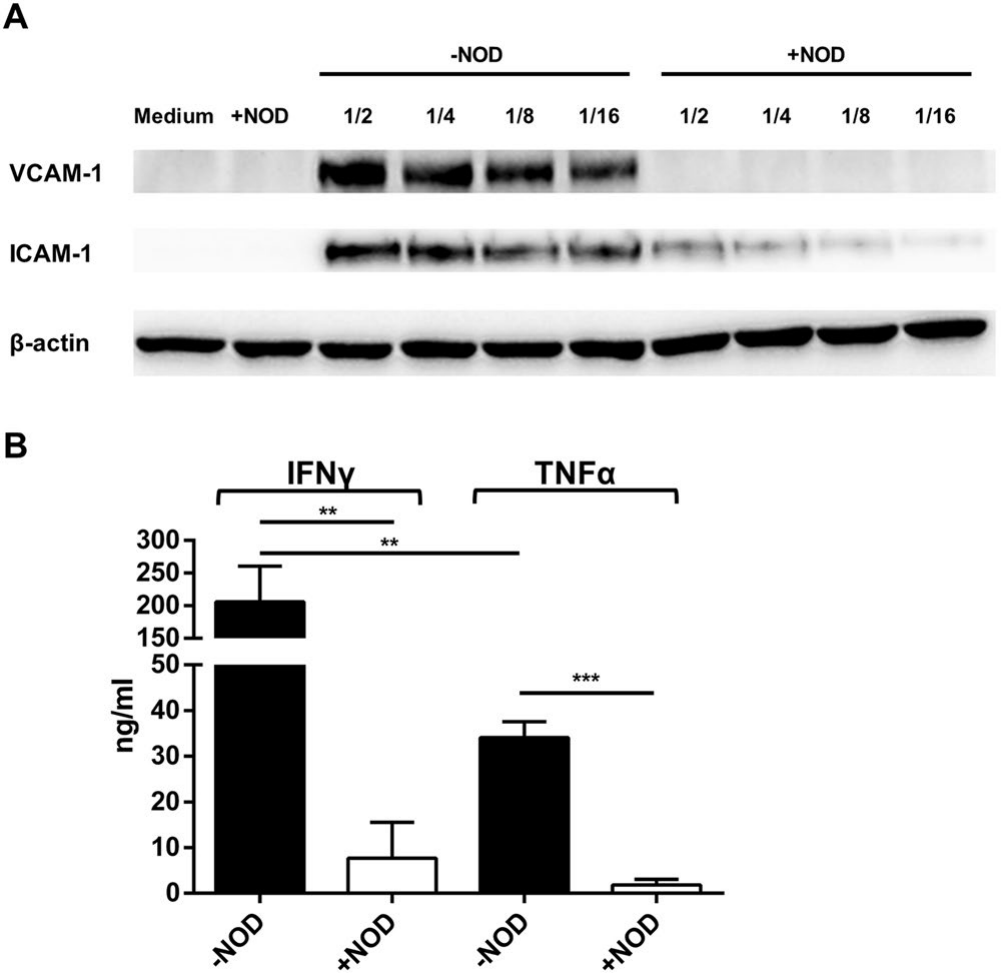
Supernatants of T-cells that are activated in the presence of NOD lose their ability to induce VCAM-1 and ICAM-1 on endothelial cells. (**A**) HUVEC were stimulated overnight by serial dilution of supernatant collected from anti-CD3/CD28 activated T-cells. Activation was performed in the presence or absence of 100 µM of NOD. VCAM-1 and ICAM-1 expression were assessed by Western-Blotting. HUVEC cultured in cell culture medium to which either or not 50 µM NOD was added served as control. Equal protein loading was demonstrated by staining for β-actin. The results of a representative blot are depicted. A total of 3 independent experiments with different HUVEC cultures were performed. Displayed are the cropped blots. The scanned full-length blots are provided in Supplementary Fig. 1. Densitometry graphs with significance marks are provided in Supplementary Fig. 2. (**B**) TNFα and IFNγ concentrations were assessed by ELISA in the T-cell supernatants described in A. Values represent mean cytokine concentration + SD from three independent experiments. Data were analyzed using two-way ANOVA followed by Sidak’s multiple comparison test. Significance was defined as *p*-value < 0.05 (**p < 0.01, ***p < 0.001) for stimulated/−NOD compared to stimulated/+NOD.

To test if the propensity of anti-CD3/anti-CD28 activated T-cell supernatants to facilitate T-cell adhesion correlates with their ability to increase VCAM-1 and ICAM-1 expression on endothelial cells, we incubated endothelial cells overnight with these supernatants. While anti-CD3/anti-CD28 activated T-cell supernatants clearly increased the expression of both adhesion molecules even when used at high (1/16) dilutions, supernatants obtained from T-cells that were activated in the presence of 100 µM of NOD did not induce VCAM-1 at all and ICAM-1 only to a little extent (Fig. 3A). We also assessed the concentration of TNFα and IFNγ in these supernatants. As depicted in Fig. 3B, activated T-cells produce approximately 6-fold more IFNγ as compared to TNFα (Mean production ng/ml ± SD 206.20 ± 0.44.30 vs. 34.04 ± 2.84, IFNγ vs. TNFα, *p* < 0,01) (Fig. 3B). When T-cells were activated in the presence of NOD both the production of IFNγ and TNFα was strongly diminished (IFNγ mean production ng/ml ± SD: 206.20 ± 44.30 vs. 7.67 ± .6.41; −NOD vs. +NOD, *p* < 0.01 and TNFα mean production ng/ml ± SD: 34.04 ± 2.84 vs. 1.84 ± 0.99; −NOD vs. +NOD, *p* < 0.001) (Fig. 3B).

### Influence of NOD on IFNγ mediated gene expression in endothelial cells

**Figure 4:**
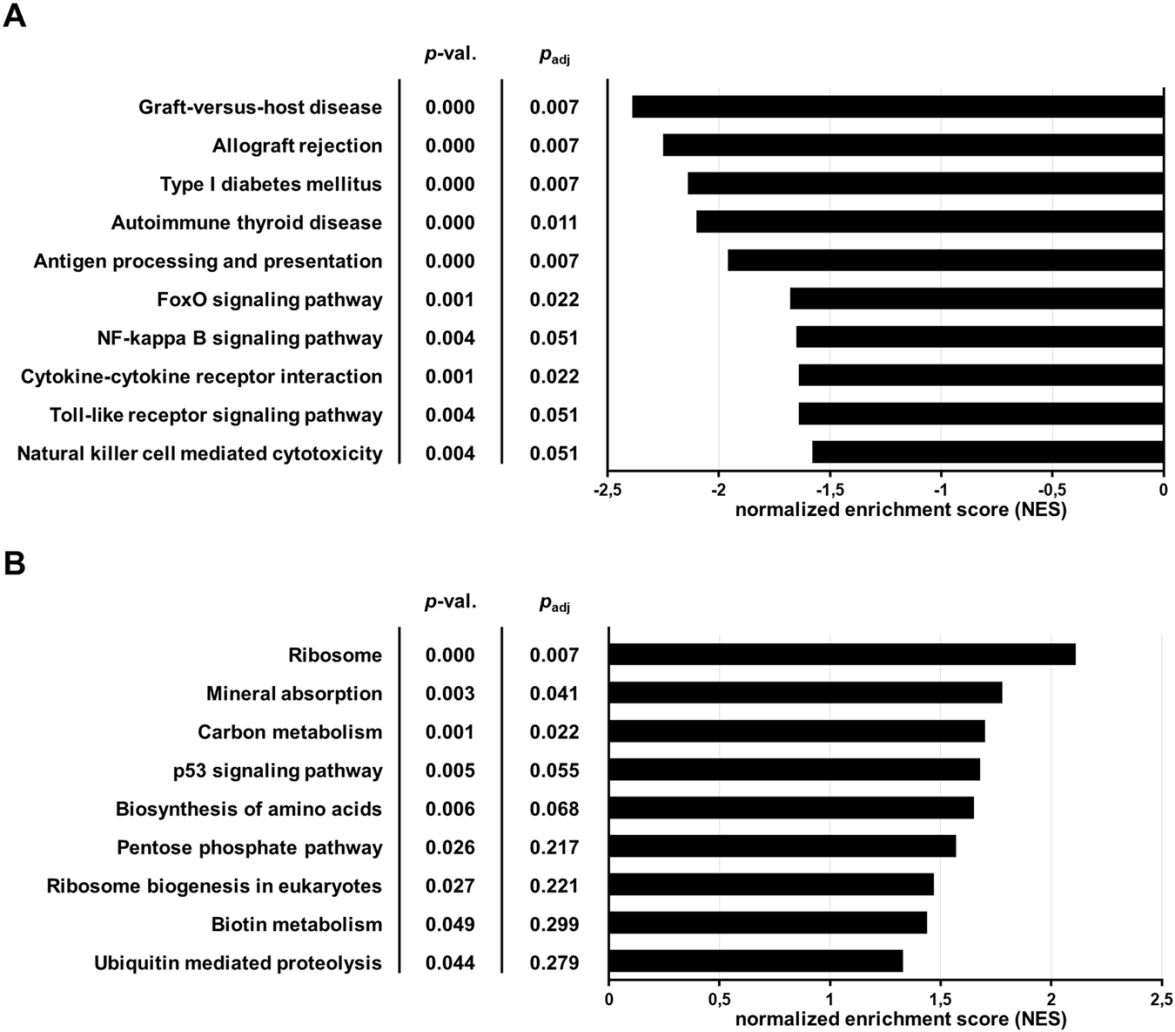
Pathway analysis by gene set enrichment. Gene expression profiling and subsequent Gene Set Enrichment Analysis was performed on IFNγ (50 ng/ml) stimulated HUVEC in the presence or absence of 50 µM of NOD. Data were analyzed as described in materials and methods. Selected enriched pathways inhibited by NOD are depicted in (**A**), while those that are upregulated in the presence of NOD are depicted in (**B**). The results are expressed as normalized enrichment score (NES) for +NOD vs. −NOD. Significance was defined as a *p*-value < 0.05 for +IFNγ/+NOD compared to +IFNγ/−NOD.

Inasmuch as the presence of IFNγ was pronounced in supernatants of activated T-cells, we subsequently tested if NOD affects IFNγ mediated gene expression in endothelial cells. To this end, genome wide gene expression profiling was performed in endothelial cells stimulated with IFNγ either in the presence or absence of 50 µM of NOD^21^. Gene set enrichment analysis (GSEA) was performed to identify pathways in the Kyoto Encyclopedia of Genes and Genomes (KEGG) database that were influenced by NOD. Enlisted in Fig. 4A is a selection of pathways that are down-regulated when endothelial cells were stimulated with IFNγ in the presence of NOD. Based on an adjusted *p*-value (*p*_adj_) for significance *p*_adj_ < 0.05, only 7 of these pathways were significantly affected. The strongest inhibitory effects of NOD were found in the Graft-versus-host disease pathway (Normalised enrichment score (NES): −2.39, *p*_adj_ = 0.007), Allograft rejection pathway (NES: −2.25, *p*_adj_ = 0.007), Type I diabetes mellitus pathway (NES: −2.14, *p*_adj_ = 0.007) and Antigen processing and presentation pathway (NES: −1.96, *p*_adj_ = 0.007). Figure 4B shows a selection of pathways that were up-regulated in the presence of NOD of which only 3 pathways remained significant after adjustment for multiple testing. At the single gene level genes belonging to MHC class II (HLA-DOA,-DPA1,-DPB1,-DRA,-DRB1,-DMA,-DMB), the MHC class II antigen-associated invariant chain (CD 74), and MHC class II transactivator (CIITA) were identified amongst the genes that were at least 2-fold down regulated by NOD. Likewise interferon regulated factor-1 (IRF-1), a number of interferon-induced proteins (IFI30, -IFI35, IFI44 and IFI44 like) and interferon-induced protein with tetratricopeptide repeats (IFIT3, and IFIT2) as well as genes that are known to be up-regulated by IFNγ, e.g. CX3CL1^23^, TAP1^24^ and, IDO1^25^ were down regulated by NOD. Yet, for a number of these genes these changes did not reach statistical significance after Bonferroni correction (Table 1).

**Table 1:** Enlisted are genes with nominal significance (*p*-value (-Log10) >1,66 and fold change (Log2) of at least 1.

Gene symbol^*^	Fold change (Log2)	*p*-value (-Log10)	*p*_adj_
CX3CL1	2,68	1,66	0,26
IDO1	2,48	2,47	0,11
HLA-DOA	1,92	2,25	0,14
CIITA	1,87	2,45	0,12
**IFI30**	1,69	3,37	0,05
IFIT3	1,67	2,43	0,12
HLA-DPA1	1,60	2,70	0,09
**HLA-DMB**	1,48	3,26	0,05
**IFI44L**	1,48	3,30	0,05
**HLA-DMA**	1,39	3,83	0,04
HLA-DPB1	1,39	2,67	0,09
**IRF1**	1,34	3,69	0,04
**CD74**	1,34	4,51	0,03
IFI35	1,30	2,35	0,13
HLA-DPB2	1,26	2,21	0,15
**HLA-DRA**	1,26	4,90	0,03
IFIT2	1,16	1,87	0,21
HLA-DRB1	1,07	2,78	0,08
**TAP1**	1,03	4,09	0,03
IFI44	1,00	3,08	0,06

^*^Gene symbols in bold depicts genes that remain significant after Bonferroni correction, underscored gene symbols represent genes with borderline significance after Bonferroni correction.

### IFNγ-mediated expression of CD74, CIITA and HLA-DR is inhibited by NOD

**Figure 5:**
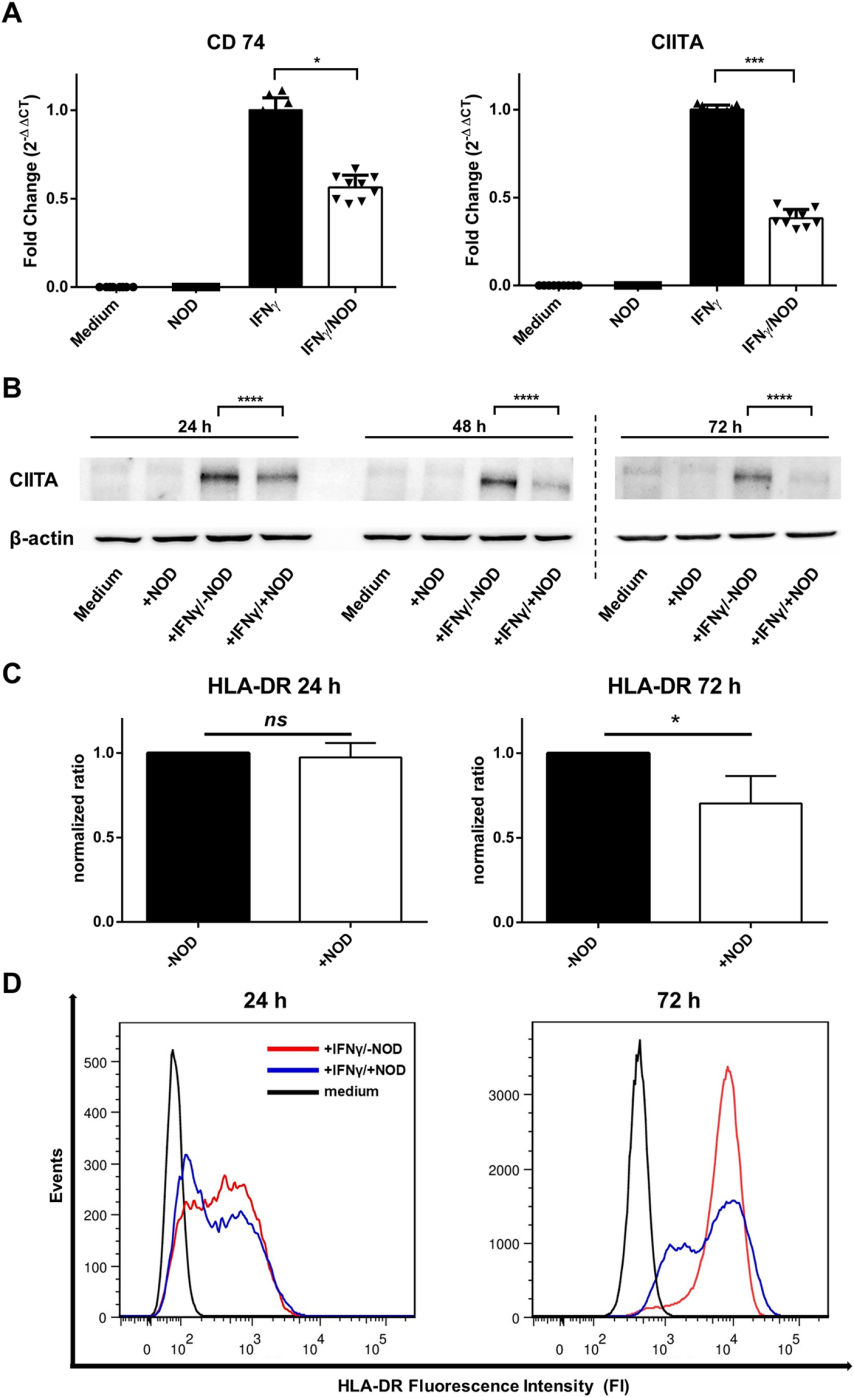
NOD inhibits the expression of CD74, CIITA and HLA-DR. (**A**) CIITA and CD74 mRNA expression was assessed by qPCR. Total RNA was isolated from HUVECs cultured in normal medium or stimulated by IFNγ (50 ng/ml). IFNγ stimulation occurred in the absence (filled bars) or presence (open bars) of 50 µM of NOD. The results of three independent experiments are expressed as the mean fold change (2^−ΔCT^) + SD. Data were analyzed using two-way ANOVA followed by Sidak´s multiple comparison test on the normalized ΔCT values. Significance was defined as *p*-value < 0.05 (*p < 0.05; ***p < 0.001) for +IFNγ/−NOD compared to +IFNγ/+NOD. (**B**) CIITA protein expression was assessed by Western-Blotting using cell-lysates obtained from HUVECs cultured for various times as described in A. The results of a representative blot are depicted, a total of 3 independent experiments with different HUVEC cultures were performed. CIITA protein expression was quantitated by means of densitometry of all independent blots. Data were analyzed using two-way ANOVA followed by Sidak´s multiple comparison test. Significance was defined as *p*-value < 0.05 for +IFNγ/−NOD compared to +IFNγ/+NOD. Equal protein loading was demonstrated by staining for β-actin. Displayed are the cropped blots. The scanned full-length blots are provided in Supplementary Fig. 3. Densitometry graphs with significance marks are provided in Supplementary Fig. 4. (**C**) HLA-DR expression was assessed by FACS analyses of HUVECs cultured for 24 or 72 hours as described in (**A**). A total of 3 independent experiments using 3 different HUVEC donors were performed. Since each donor differed in the extent by which HLA-DR was up-regulated by IFNγ, for each donor the mean HLA-DR fluorescence intensity (MFI) was normalized for IFNγ stimulation. The results are expressed as mean normalized ratio + SD, i.e. (MFI HLA-DR +NOD/MFI HLA-DR −NOD) with (MFI HLA-DR −NOD/MFI HLA-DR −NOD) as 1. (*p < 0.05; ns: not significant). (**D**) The results of a representative experiment are shown as histograms. Red histogram: HUVEC stimulated with IFNγ only, blue histogram: HUVEC stimulated with IFNγ and NOD, black histogram: HUVEC not stimulated.

Since gene expression profiling data suggested that NOD might affect MHC class II expression in endothelial cells, we first assessed by TaqMan PCR to what extent CD74 and CIITA mRNA expression was influenced when endothelial cells were stimulated for 24 hrs with IFNγ in the presence or absence of NOD. As depicted in Fig. 5A both CD74 and CIITA mRNA were strongly induced by IFNγ. In the presence of NOD the up-regulation of CD74 and CIITA mRNA was significantly blunted (Fig. 5A). The diminished CIITA mRNA expression translated in a reduced expression of CIITA protein, which became more pronounced after 72 hrs (Fig. 5B). In line with this, it was found that IFNγ mediated HLA-DR surface expression was reduced on endothelial cells when stimulated in the presence of NOD (At 24 hrs % positive cells: 68 vs 58, MFI: 365 vs. 281; at 72 hrs % positive cells: 92 vs 57, MFI: 6706 vs. 4762, −NOD vs. +NOD) (Fig. 5C).

## Discussion

Even though current state-of-the-art immunosuppressive strategies to a large extent have contributed to the success of contemporary transplantation medicine, they failed to interrupt the insidious process causing chronic allograft loss. While CNI based immunosuppressive therapies are highly efficacious in preventing acute T-cell mediated rejection, they are also associated with significant adverse effects such as hypertension and nephrotoxicity^26^. Attempts to minimize or wean patients off of CNI have shown that improvement in renal function is often obtainable but only at the expense of increased alloimmune reactions^27,28^. The development of a CNI-based, long-term, maintenance-immunosuppressive drug regimen with improved long-term tolerability is therefore a highly desirable endeavour.

We and others have previously reported that N-acyl dopamine derivatives (NADD) harbour biological properties that might be useful for preventing transplantation associated injury^29–31^. As such they limit cold inflicted^32^ and ischemia reperfusion-injury^33^ and modulate inflammation through inhibition of NFκB^21,34^. We also have reported that NOD, a short synthetic NADD, transiently inhibits T-cell activation and demonstrates synergy with CNI in suppressing T-cell activation^22^.

The results presented herein are in good agreement with our previous results that NOD inhibits anti-CD3/antiCD28 mediated T-cell activation. This is reflected by a reduction in cytokine production and a diminished expression of VLA-4 and LFA-1 on the cell surface of T-cells. Because VLA-4 can mediate both rolling and arrest of T-cells on endothelial cells^35^, this explains why T-cells that are activated in the presence of NOD display decreased adherence to endothelial cells despite cytokine mediated upregulation of VLA-4’s major counterreceptor VCAM-1. The reduced LFA-1 - ICAM-1 interaction is likewise contributing to the diminished adhesive properties of such activated T-cells. In addition to VCAM-1 and ICAM-1 also junctional adhesion molecules (JAM-A, -B and -C) have been implicated in regulating leucocyte extravasation by affecting different steps in this process through their interaction with endothelial cells. Endothelial JAM-A and JAM-C interact with the ß2 integrins LFA-1 and Mac-1 (CD11b/CD18) to sustain firm adhesion and transmigration^36,37^. Although in the present study transmigration was not assessed, our data do suggest that extravasation of T-cells would be impaired as a consequence of a reduced CD18 expression if T-cell activation occurs in the presence of NOD.

In addition to its effect on T-cell activation, gene expression profiling revealed that NOD also affects the transcriptome of IFNγ stimulated endothelial cells. Significant normalised enrichment scores were amongst others found in the Graft-versus-host disease pathway, Allograft rejection pathway, Type I diabetes mellitus pathway and Antigen processing and presentation in all of which MHC class I and II, as well as IFNγ have been implemented. This not necessarily indicates that NOD *per se* interferes with IFNγ signalling, since the influence of NOD on other pivotal mediators of these pathway have not been studied. However, because both MHC Class II and IFNγ are of pivotal importance in terms of tissue immunogenicity allograft rejection, the influence NOD on IFNγ mediated MHC class II expression was studied in more detail. Although other pathways were also influenced by NOD, e.g. ribosome, p53 mineral absorption, they were not further studied because an apparent link with tissue immunogenicity or allograft rejection was lacking.

MHC class II proteins bind microbial peptides in an endosomal compartment and present them to T-cells as part of immune surveillance. While in the endoplasmic reticulum the hydrophobic peptide-binding groove in MHC class II is protected by the invariant chain CD74^38^, in the late endosomal compartment CD74 is degraded leaving class II-associated invariant chain peptides (CLIP) in the peptide binding groove^39,40^. HLA-DM catalyzes CLIP dissociation, stabilizes empty MHC class II proteins, and enables rapid binding of microbial peptides^41^. Since at the single gene level, NOD inhibits the transcription factor for MHC class II expression, i.e. CIITA, as well as HLA-DR, HLA-DM and CD74 expression, our data suggest that NOD might impair MHC class II presentation. Yet, endothelial cells are not endowed with a professional antigen presentation function as they fail to express classical co-stimulatory molecules. Nonetheless increased MHC class I and II expression on endothelial cells may render the allograft more susceptible to alloantigen-dependent vascular damage and to the development and progression of transplant vasculopathy. It needs to be assessed if and to what extent MHC expression and antigen presentation on professional antigen presenting cells is affected by NOD.

The present study has some limitations that should be addressed. In paticular, the mechanism by which NOD inhibits IFNγ mediated gene expression is currently unclear. While the catechol structure provides NOD with strong antioxidant properties, the hydrophobic fatty acid of NOD might allow its passage across membranes to access different intra-cellular compartments and change the redox milieu within these compartments. As such NOD may impair oxidative protein folding in the endoplasmic reticulum (ER) and consequently induce the unfolded protein response (UPR)^42,43^, or inhibit redox dependent transcription factors in the nucleus, e.g. NF-κB, AP-1 and NFAT^22^. Cross-talk between IFNγ signalling and the UPR may partly underlie the inhibitory effect of NOD as the UPR is associated with transient inhibition of mRNA translation^44^. Alternatively, changes in the redox milieu of the cell nucleus may impair epigenetic processes required for gene transcription^45^. Indeed, gene expression profiling studies on endothelial cells have shown that histone acetyl transferase expression was affected by NOD. Even though all the above mentioned mechanisms may explain the *in vitro* effects of NOD, further *in vivo* experiments are warranted to elucidate which of these putative mechanisms also occur *in vivo*. The NOD concentrations used in the current study were based on previous *in vitro* studies^22,43^, yet the a fast blood clearance, predominantly via renal and hepatobiliary elimination^46^ does not allow plasma NOD concentrations to reach the *in vitro* concentrations used in our current study. Despite this short-coming, *in vivo* studies demonstrated that NOD is highly efficacious in the treatment of brain dead donors on early post-transplant renal and heart function^47,48^, in ameliorating inflammation in ischemic acute kidney injury^33^ and in attenuating the development of transplant vasculopathy in rat aortic allografts^49^. Nonetheless, it remains to be elucidated to what extent the *in vivo* efficacy of NOD is mediated by its ability to inhibit IFNγ mediated gene expression.

In conclusion, this study provides further evidence for the immunosuppressive properties of NOD on T-cell activation. Not only the present study suggests a potential clinical use for NOD to limit the development of IFTA in allograft recipient, but it also warrants further *in vivo* testing in relevant models for transplantation.

## Methods

### Cell culture

Human umbilical vein endothelial cells (HUVECs) were isolated from fresh umbilical cords as described previously^50^. Confluent monolayers were passaged by trypsin 0.025%/ EDTA 0.01% and mostly used for experiments after 2–3 passages. Umbilical cords were obtained from the Department of Obstetrics, University Medical Center Mannheim, after written informed consent of the patients. The study was approved by the institutional ethics committee (Medizinische Ethikkommission II der Medizinischen Fakultät Mannheim (No. 2015-518N-MA)) and carried out in accordance with the relevant guidelines and regulations.

Human peripheral blood mononuclear cells (PBMC) were isolated from healthy adult volunteer donors by Ficoll-Hypaque density gradients (GE Healthcare Bio-Sciences AB, Uppsala, Sweden). T-cells were purified using a Pan T-cell Isolation Kit (Miltenyi Biotec Inc., Auburn, CA, USA), according to the manufacturer’s instructions, seeded in 24 well plates (10^6^ cells/ml in RPMI 1640/ 10% FBS (PAA Laboratories GmbH, Pasching, Austria)/ 1% penicillin and streptomycin (Sigma-Aldrich, St. Louis, USA) and stimulated for 3 days with 15 µl/ml Streptamer® anti-CD3/anti-CD28 Premix (IBA GmbH, Göttingen, Germany) in the presence or absence of 100 µM NOD (Novaliq GmbH, Heidelberg, Germany). T-cells were harvested by FACS and supernatants were used for assessment of TNF-α and IFNγ and their ability to induce VCAM-1 und ICAM-1 expression on endothelial cells. The propensity to adhere-, or facilitate adhesion to endothelial cells was tested for T-cells and supernatants.

### Adhesion assay

Confluent HUVEC monolayer were stimulated for 24 hours with either TNFα (10 ng/ml) (PeproTech, Hamburg, Germany) or IFNγ (50 ng/ml) (Sigma-Aldrich, St. Louis, USA) or left in normal culture medium. In some experiments HUVEC monolayers were incubated with a serial dilution (1/2 to 1/16) of T-cell supernatants. T-cells were stimulated as described above, labelled with CytoTell Green (AAT Bioquest, Sunnyvale, USA) as recommended and added to the endothelial monolayers for 1 hour in a concentration of 10^6^ T-cells per well. Before addition of labelled T-cells, the HUVEC monolayers were washed with PBS to remove the stimulating cytokines. Non-adherent T-cells were removed by washing and the remaining cells were lysed in 10 mM Tris, 2% SDS. Fluorescence was measured on a Tecan Spark 10 M with the appropriate filters (Tecan Group, Männedorf, Switzerland). All experimental conditions were performed in triplicate. Results are expressed as mean relative fluorescence units (RFU) ± SD.

### Western-blotting

Western-Blotting was performed as described before^21^. Samples (20 µg protein extract) were separated on 10% SDS-polyacryamide gels followed by semi-dry blotting onto PVDF membranes (Roche, Basel, Switzerland). Membranes were blocked for 1 hour at room temperature in tris buffered saline (TBS) (Bio-Rad, Hercules, USA) and incubated overnight at 4 °C with one of the following primary polyclonal antibodies: anti-VCAM-1, anti-ICAM-1 (both from R&D Systems, Wiesbaden, Germany) or anti-CIITA (Santa Cruz Biotechnology, Dallas, USA). Hereafter, the membranes were thoroughly washed with TBS 0,1% Tween-20 and incubated with the appropriate horseradish peroxidase conjugated secondary antibody (Jackson ImmunoResearch, Baltimore, USA). Proteins were visualized using enhanced chemo luminescence technology (Pierce, Rockford, USA). To confirm equal protein loading, membranes were stripped and re-probed with monoclonal anti-β-actin (Santa Cruz Biotechnology, Dallas, USA) antibody.

### ELISA

For assessment of IFNγ and TNFα concentrations in supernatants of anti-CD3/anti-CD28 activated T-cells commercially available ELISA systems were used (R&D Systems, Minneapolis, USA; Catalog No. DIF50 and DTA00C)

### RNA Isolation and gene expression profiling

Total RNA was prepared using Trizol reagent (Ambion, Carlsbad, USA). RNA quality was confirmed by capillary electrophoresis on an Agilent 2100 bioanalyzer. For the microarray experiments HUVEC were obtained from three different donors and for each donor stimulated in T25 culture flasks with IFNγ (50 ng/ml) alone or with the combination of IFNγ and NOD (50 µM). Sample preparation and processing was performed according to the Affymetrix GeneChip Expression Analysis Manual (http://www.Affymetrix.com) as described extensively elsewhere^21^.

### Quantitative PCR

For qPCR 1 µg of total RNA was reverse-transcribed into cDNA using the 1st Strand cDNA Synthesis Kit. cDNA was diluted in 20 µL DEPC-treated water and stored at −20 °C until use. qPCR was performed on a StepOne™ Real-Time PCR System (Applied Biosystems, Foster City, USA) using TaqMan Fast Advanced Master Mix (Applied Biosystems, Foster City, USA) and the following TaqMan probes (all from Applied Biosystems, Foster City, USA): HMOX1 (HO-1, ID: Hs 01110250_m1), CIITA (ID: Hs00172106_m1) and CD74 (ID: Hs00269961_m1). Samples were run under the following conditions: initial denaturation for 10 min at 95 °C followed by 40 cycles of 15 s at 95 °C and 1 min at 60 °C. The levels of gene expression in each sample were determined with the comparative cycle threshold method. PCR efficiency was assessed from the slopes of the standard curves and was found to be between 90% and 100%. Linearity of the assay could be demonstrated by serial dilution of all standards and cDNA. All samples were normalized for an equal expression of β-actin.

### FACS analysis

For FACS analysis HUVEC were incubated with anti-HLA-DR PE conjugated antibody (BD-Biosciences, Heidelberg, Germany). Anti-CD3/anti-CD28 activated T-cells four color-staining was performed using the following monoclonal antibodies: anti-CD49d-PE, anti-CD29-APC, anti-CD11a-BV421 and anti-CD18-FITC (all purchased from BD Biosciences, Heidelberg, Germany). For each antibody optimal dilutions were assessed by serial dilutions. Stained samples were measured on a BD FACSlyricTM Flow cytometer using both negative and fluorescence-minus-one controls. Compensation was performed with BD compBeads (BD Biosciences, Heidelberg, Germany). Data analysis was performed using FlowJo software version 5.2 (FlowJo LLC, Ashland, USA).

### Statistical analysis

A Custom CDF Version 21 with ENTREZ based gene definitions was used to annotate the arrays^51^. The raw fluorescence intensity values were normalized applying quantile normalization and RMA background correction. One-way ANOVA was performed to identify differential expressed genes using a commercial software package SAS JMP10 Genomics, version 6, from SAS (SAS Institute, Cary, USA). A false positive rate of a = 0.05 with FDR correction was taken as the level of significance. Gene Set Enrichment Analysis (GSEA) was used to determine whether defined lists (or sets) of genes exhibit a statistically significant bias in their distribution within a ranked gene list using the software R v3.4.0^52^ and RStudio: Integrated development environment for R (RStudio Boston, USA). The fgsea package^53^ was used to determine whether defined lists (or sets) of genes exhibit a statistically significant bias in their distribution within a ranked gene list. Pathways belonging to various cell functions such as cell cycle or apoptosis were obtained from public external databases (KEGG, http://www.genome.jp/kegg).

Statistical analysis for the Adhesion Assay was performed by two-way anova followed by Sidak´s multiple comparison test. Quantitative RT-PCR and Adherence Assays were analysed using a two-way ANOVA and Sidak´s multiple comparison test. ELISA data were analysed using two-tailed unpaired t-test. For Western-Blotting average optical densities (OD) of bands of 3 independent experiments were assessed using ImageJ 1.46 and subsequently two-way ANOVA and Sidak’s multiple comparison test on OD ratio of protein of interest/housekeeping protein or phosphorylated/total protein. A *p*-value of less than 0.05 was considered significant.

## Supplementary information

Supplementary Material

## Data Availability

The datasets generated during and/or analysed during the current study are available from the corresponding author on reasonable request.
