# Supplementary material for: N-Octanoyl-Dopamine inhibits cytokine production in activated T-cells and diminishes MHC-class-II expression as well as adhesion molecules in IFNγ-stimulated endothelial cells
Source: Sci Rep. 2019 Dec 18;9:19338. doi: 10.1038/s41598-019-55983-1 (PMC6920350; doi:10.1038/s41598-019-55983-1)
Supplement: Supplementary file 1 — Supplementary Material [file 41598_2019_55983_MOESM1_ESM.docx]

**Supplementary Information**

**N-Octanoyl-Dopamine inhibits cytokine production in activated T-cells and diminishes MHC-class-II expression as well as adhesion molecules in IFNγ-stimulated endothelial cells**

Björn B. Hofmann^1^; Nicolas Krapp^1^; Yingchun Li^1^; Carolina de la Torre^2^; Marloes Sol^3^; Jana D. Braun^1^; Matthias Kolibabka^1^; Prama Pallavi^1^; Bernhard K. Krämer^1^; Benito A. Yard*^1^ and Anna-Isabelle Kälsch^1^

^1^Department of Nephrology, Endocrinology and Rheumatology, Fifth Department of Medicine, Medical Faculty Mannheim, University of Heidelberg, Mannheim, Germany, ^2^Center of Medical Research, Medical Faculty Mannheim, University of Heidelberg, Mannheim, Germany, ^3^Department of Medical Biology and Pathology, University Medical Center Groningen, Groningen, Netherlands

*Corresponding author: Prof. Dr. Benito A. Yard

Email: benito.yard@medma.uni-heidelberg.de

Address: Universitätsklinikum Mannheim, V. Medizinische Klinik, Theodor-Kutzer-Ufer 1-3, 68167 Mannheim, Germany


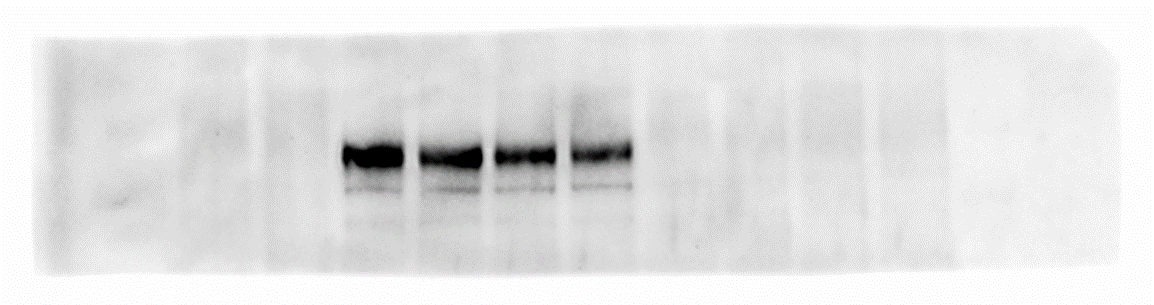




**B**

**A**

**ICAM-1**

**VCAM-1**





**β-actin**

**C**

**Supplementary Figure 1: Full length blots of figure 3A.**

Full length blots of VCAM-1 (A), ICAM-1 (B) and β-actin (C). The arrows point to the relevant protein bands.


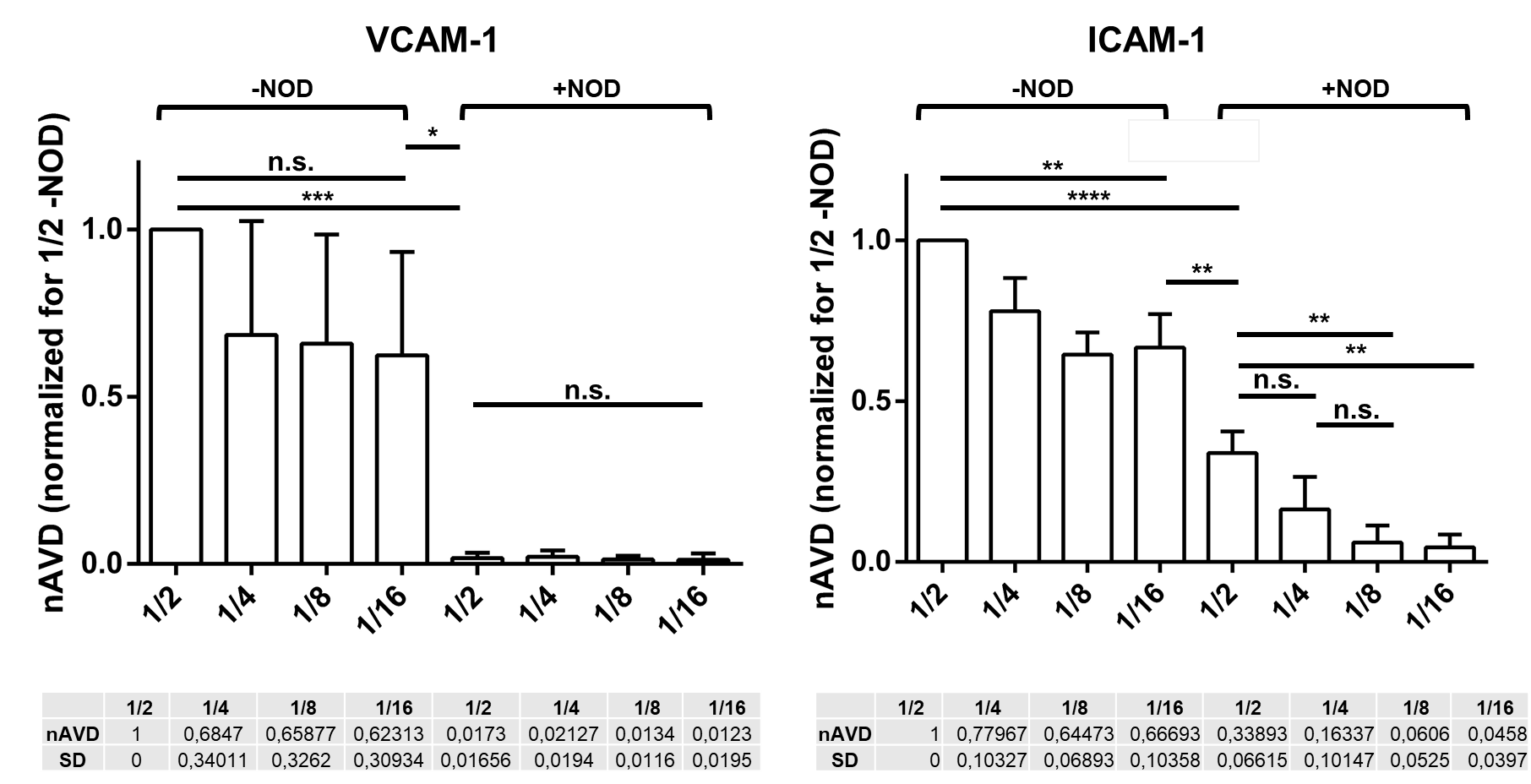


**Supplementary Figure 2: Densitometry for Western Blott 3A**

Density was evaluated using imageJ Software. After normalisation for β-actin the normalized average Density (nAVD) of the 3 independent experiments was calculated. Normalization for ½ -NOD. Data were analysed using two-way ANOVA followed by Sidak´s multiple comparison test. A p-value < 0.05 was considered to be significant. (*p≤0.05; **p≤0.01; ***p≤0.001; ****p≤0.0001)

**

**

**A**

**B**

**CIITA**

**

**

**C**

**β-actin**

**

**

**D**

**CIITA**

**
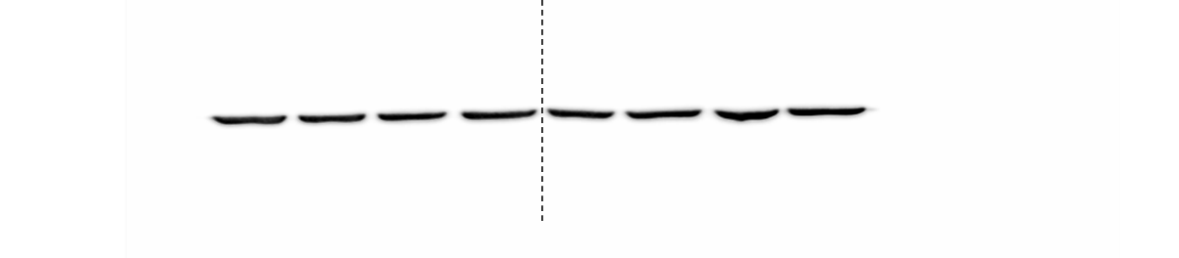
**

**β-actin**

**Supplementary Figure 3: Full length blots of figure 5B.**

Full length blots of CIITA (A+C) and β-actin (B+D). The arrows point to the relevant protein bands. Notice that the upper portion of the second gel was divided before blotting and CIITA staining (displayed in C, four bands), whilst the lower portion, displayed in D, remained unseparated for β-actin staining (eight bands).

**
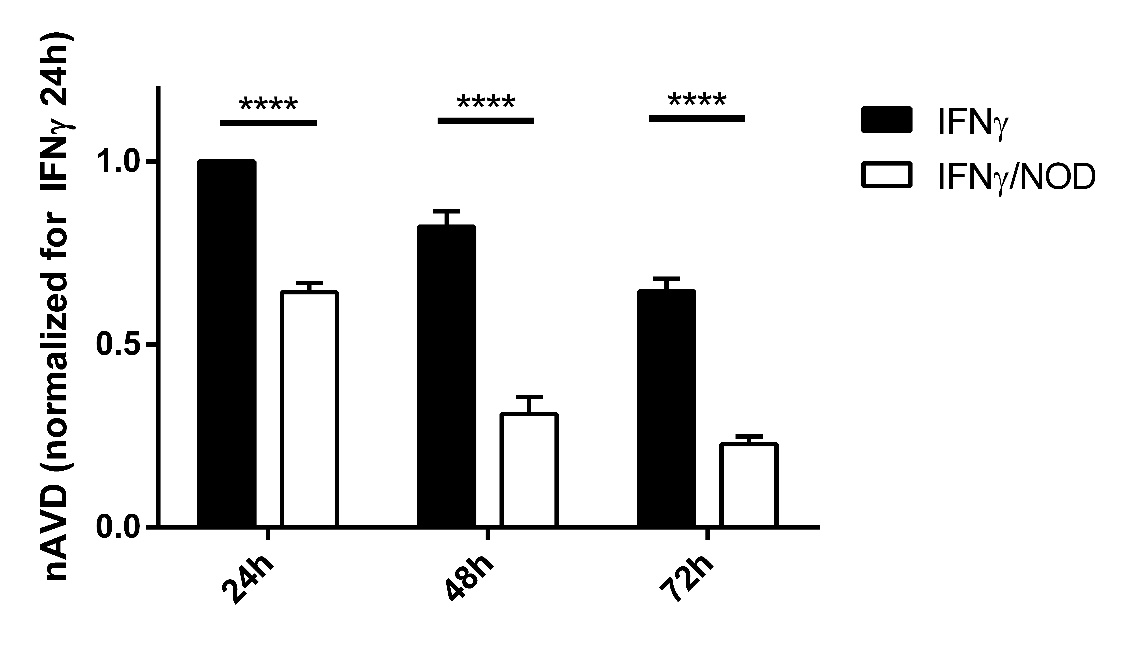
**


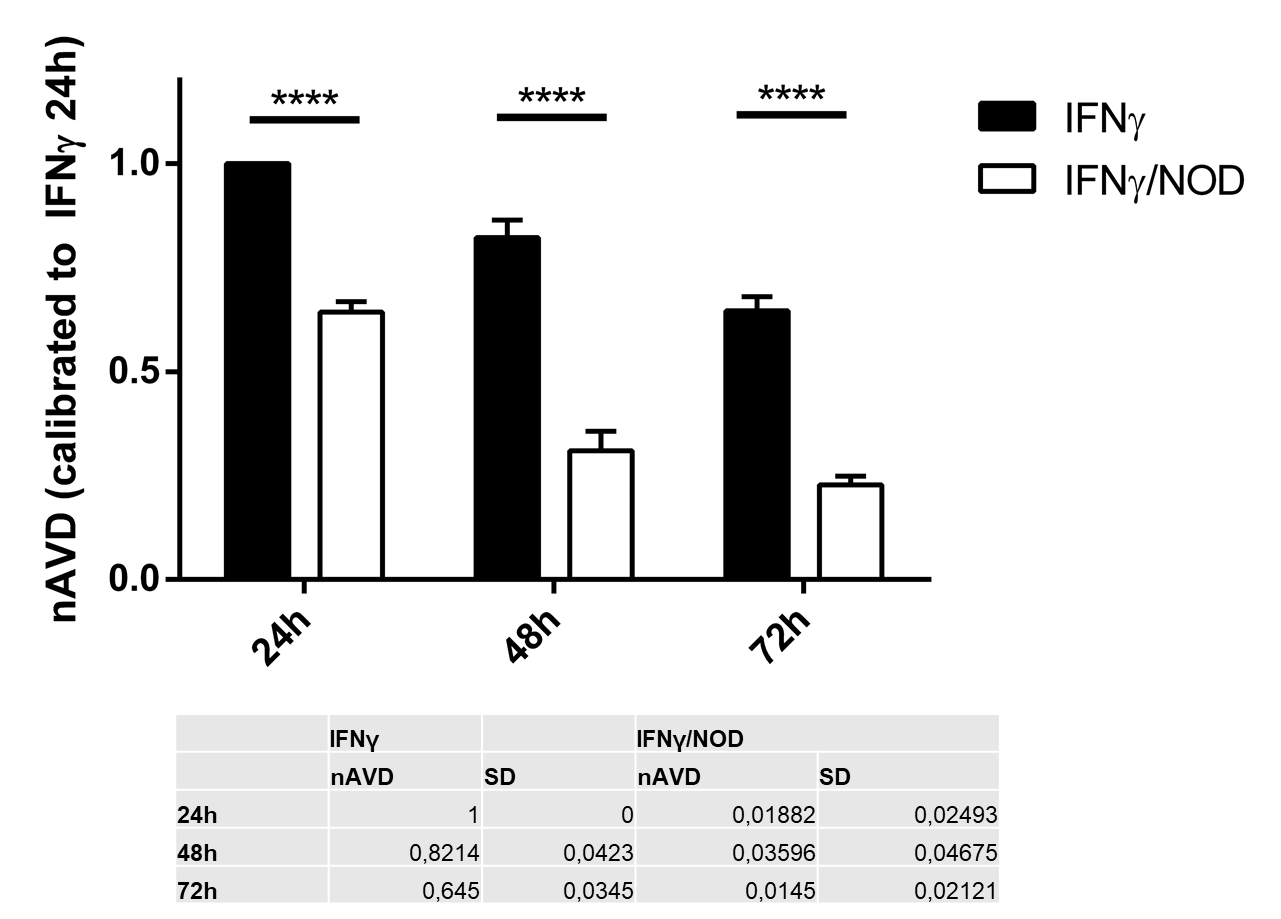


**Supplementary Figure 4: Densitometry for Western Blott 5B**

Density was evaluated using imageJ Software. After normalization for β-actin the normalised average density (nAVD) of the 3 independent experiments was calculated. Normalization for 24h IFNγ. Data were analysed using two-way ANOVA followed by Sidak´s multiple comparison test. A p-value < 0.05 was considered to be significant. (****p≤0.0001)
